# On the Continuous Use of Blue Mass in Small Doses

**Published:** 1890-04

**Authors:** 


### PEPPER (W.) ON THE CONTINUOUS USE OF BLUE MASS IN SMALL DOSES.

---

The patient was a man of large frame, about fifty-five years of age, whose occupation or habits prevented him from getting sufficient sleep, as he rose each morning by 4 or 4:30, although he did not retire until 11 P. M. Evident signs of heart failure began to show themselves about a year ago, with dyspnœa on exertion, difficulty in going up stairs, of lifting weights, increasing œdema and albumen in the urine without casts. He had been treated in various ways, chiefly by iron in conjunction with diuretics, as acetate of ammonia or nitrate of potash. The symptoms steadily increased until the œedema invaded the trunk and genitals, and he was almost confined to his room. When he came under treatment repeated trial was made with similar remedies, but finding no good result, and that the digestive system was in fairly good condition, the following pill was ordered:

R Mass. hydrargyri
Pulv. digitalis
Cinchonidiæ sulph. - - - - aa gr. xl
Fiat mass. et div. in pil. No. xl.
Sig: One pill three times a day.

These pills were begun November 10th, and were continued regularly until November 22nd, by which time the full number had been taken. He was also ordered to remain in bed until 7

o'clock each morning, thus securing at least eight hours' rest. During the day he was directed to lie down for one hour.

The only appreciable action of the remedy was a steadily improving tone of cardiac action with increased secretion of urine, with diminished proportion of albumen, and progressive decrease of œdema. By November 22nd all the symtoms had disappeared, the urine was free from albumen, and the œdema was entirely gone. There had been no purgation, and no evidence of mercurial action. The change in his appearance was extraordinary, as he seemed shrunken away, showing that the entire body had been inflated with serum. He felt weak, but the only remedy ordered was an ounce of whisky twice daily. Upon this he has rapidly regained his strength, and now seems in very good condition and ready to return to work.

I have in some cases of general œdema, both with weak heart and with organically diseased heart, given the above combination for much longer periods than in this case, and with remarkably good results, but in other cases it becomes necessary to suspend very soon, chiefly owing to gastric disturbance. If the mouth is frequently washed with a solution of chlorate of potash, there does not seem much danger of ptyalism; but a constant close watch for this should be kept up. The remedies should always be joined with carefully regulated hygiene and diet.—*Med. Mag.; Medical Analectic and Epitome.*

